# Modeling successive birth interval of women in Ethiopia: application of parametric shared frailty and accelerated failure time model

**DOI:** 10.1186/s12905-021-01190-y

**Published:** 2021-01-30

**Authors:** Nuru Muhammed Mustefa, Denekew Bitew Belay

**Affiliations:** 1 Department of Statistics, Woldia University, Woldia, Ethiopia; 2 Department of Statistics, Bahir Dar University, Bahir Dar, Ethiopia

**Keywords:** Birth interval, AFT model, Frailty model

## Abstract

**Background:**

Both short and long birth intervals are associated with many risk factors and about 29% of births are short birth intervals in Ethiopia. The purpose of this study is to model the birth intervals of adult women aged 15–49 years using accelerated failure time and shared frailty models in order to analyze the birth intervals of Ethiopian women.

**Methods:**

The data was obtained from the 2016 Ethiopian Demographic and Health Survey (EDHS). Accelerated failure time with different baseline and shared frailty models are used for the analysis to identify important demographic and socio-economic factors affecting the length of birth intervals and correlates of the birth intervals respectively.

**Results:**

The data consists of 9147 women, of which about 7842 (85.5%) are closed interval and the rest of 1323(14.5%) are open interval. Accelerated failure time (AFT) result revealed that women education level, husbands education level, age at first birth, marital status, religion and family wealth index are significant factors affecting birth interval of women in Ethiopia.

**Conclusion:**

Women with closely spaced births tend to have larger family sizes when compared with women with longer inter-birth interval. Longer successive birth interval tends to reduce the total fertility rate of women. Furthermore, improvements in socio-economic status and level of education of women associate with reduced fertility, improved maternal and child wellbeing, and longer birth interval.

## Background

High fertility is defined as a total fertility rate of 5.0 or higher. Total fertility rate represents the average lifetime births per woman implied by the age-specific fertility rates prevailing in one historical period [3]. Birth of the first child is the first visible sign of fertility because it marks a woman’s transition to motherhood. It has a significant role in individual women’s life because of its direct relationship with fertility. The age at which child bearing begins in the absence of any active fertility control affects the number of children a woman bears throughout her reproductive period [15]. In sub-Saharan Africa where contraceptive use is relatively low, age at first birth affects the number of children a woman will have [19].

In the societies where births are confined to marriage, reproduction starts from the onset of effective marriage, and the birth interval following effective marriage depends on the demographic characteristic of women at the earlier stages of married life [16]. Ever since the international conference on population and development (ICPD) in 1994, fertility patterns in the world have changed dramatically leading to a world with quite diverse child bearing patterns [24].

Birth interval refers to the time length between two successive live births and longer birth interval allow full gestation and growth of next pregnancy [22]. Births too close together may associate with schizophrenic offspring and during and after pregnancy complications by [23]. Both short and long birth intervals are associated with many risk factors and about 29% of births are short birth intervals in Ethiopia [6].

A Latin American study revealed that short birth intervals adversely affect mothers’ health and chances of survival of their children. Shorter birth intervals (less than 24 months) increased maternal risks and it also led to several serious outcomes for neonates [5].

In addition to many health implications, closely spaced birth intervals inflates population growth and challenges in many development efforts for a given country. It distracted women from becoming productive members of society. Moreover, the limited resources the family invests for caring to the new born will cause the other children to receive inadequate share of the resources. Differences in a country’s fertility levels can be attributed to the differences in the length of the reproductive life of women and differences in experiences of women that cause them to conceive conducted by [21].

According to the 2007 population census of Ethiopia, the annual population growth rate within 1994–2007 was estimated to be 2.6%. Studying the effect of various socio-economic and demographic factors affecting birth interval is essential to formulate a policy that motivates people towards longer birth interval [10].

Birth history analysis provides useful information about reproduction and family formation. Further, fertility depends not only on the decision of couples but also on socio-economic, demographic, health-related, tradition-related, and emotional factors. Fertility factors affect child spacing and birth intervals reveal reproduction patterns. Analysis of the childbearing process reveal about the dynamics of fertility transitions [20].

Human fertility is a function of a variety of factors. The factors vary from place to place depending on specific conditions conducted by Lindstrom and Kiros [13] and Yohannes et al. [26]. Total fertility rate could be decreased by increasing the age at marriage [10].

## Methods

The data for this study was extracted from the dataset of Ethiopian Demographic and Health Survey (EDHS, 2016).The Ethiopian Demographic and Health Survey (EDHS, 2016) which is part of worldwide DHS project and the fourth survey belonging to the Central Statistical Agency (CSA) collected from18 January 2016 to 27 June 2016. In this analysis, successive birth Interval which refers to the age gap between successive children for each mother measured in months was used as a dependent variable.

Parametric shared and accelerated failure model and Kaplan Meier’s plot were applied to analyse the data. Kaplan–Meier was used to see the differences in birth interval and life table method was applied to include censored observation introduced by Kleinbaum and Klein [12]. The accelerated failure time model which makes inferences about the underlying risk of observations on the timing of events is described with the equation defined by Klein and Moeschberger [11] is

$$S\left( {t/X} \right) = S_{0} \left\{ {t*{\text{exp}}\left( {\alpha^{\prime}X} \right)} \right\}$$

Here we can consider the log-scale of the AFT model with respect to time given analogous to the classical linear regression approach. In this approach, the natural logarithm of the survival time Y = log (T) is modeled. This is the natural transformation made in linear models to convert positive variables to observations on the entire real line. A linear model is assumed for Y;

$${\text{Y}} = {\text{log }}(T) = \mu + \alpha^{\prime}X + \delta \varepsilon$$

where $\alpha$’ = ($\alpha$1, $\alpha$2… $\alpha$p) is a vector of regression coefficients, $\mu$ = intercept, $\delta$ = is scale parameter and, $\varepsilon$ = is the error distribution assumed to have a particular parametric distribution.

Statistical models and methods proposed to model failure time data assume that the observations are statistically independent of each other. However, this does not hold in many applications. The concept of frailty provides a suitable way to introduce random effects in the model to account for association and unobserved heterogeneity.

Estimation of the frailty model can be parametric or semi-parametric. In the former case, a parametric density is assumed for the event times, resulting in a parametric baseline hazard function. Estimation is then conducted by maximizing the marginal log-likelihood [17].

The dependent variable for this study was whether or not birth had occurred during the interval. The case was coded 0 if the event (i.e. birth) did not occur and 1 if it happened. For all cases that were censored by the year of interview, the observations were coded 0 on the dependent variable.

## Results

Descriptive analysis was conducted to get information about the distribution of the variables. The data consisted of 9147 women of which about 7842 (85.5%) were closed interval and the rest 1323(14.5%) were open interval. Summary of results of socio-economic and demographic variables of this study are presented in Table 1.

**Table 1:** Distribution of important socio-economic and demographic characteristics

Covariate/factor	Category	Event (%)	Censored	Total
Mother age	15–19	310(87%)	46	356
	20–24	1597(85.6%)	269	1866
	25–29	2326(85%)	412	2738
	30–34	1705(85.4%)	292	1997
	35–39	1287(88.9%)	197	1484
	40–44	448(84.4%)	83	531
	45–49	151(86.3%)	24	175
Residence	Urban	1427(84.8%)	255	1682
	Rural	6397(85.7%)	1068	7465
Religion	Orthodox	2248(85%)	395	2643
	Catholic	53(86.9%)	8	61
	Protestant	1378(86.4%)	247	1595
	Muslim	4013(85.6%)	674	4687
	Traditional	73(79.3%)	19	92
	Other	59(85.5%)	10	69
Women education	No education	5601(95.6%)	255	5856
	Primary	1674(73.2%)	614	2288
	Secondary and above	549(54.7%)	454	1003
Husbands education	No education	4120(99.5%)	22	4142
	Primary	2554(79.3%)	666	3220
	Secondary and above	1150(64.6%)	635	178
Type of birth	Single birth	7571(85.2%)	1312	8883
	Multiple births	253(95.8%)	11	264
Women occupation	Unemployed	4678(85.8%)	777	5455
	Employed	3146(85.25%)	546	3692
Husbands occupation	Unemployed	764(84.9%)	136	900
	Employed	6515(85.5%)	1107	7622
Wealth index	Poor	4912(85%)	863	5775
	Middle	1231(84%)	235	1466
	Rich	1681(88.2%)	225	1906
Marital status	Single	701(89.9%)	78	779
	Married	4888(85%)	856	5745
	Widowed	595(83.1%)	121	716
	Divorced	928(86.8%)	264	1069
	Separated	712(84.9%)	126	838
Age of women at 1st birth	0= ≤ 15	1057(96.6%)	40	1106
	1 = 16–20	4809(88%)	654	5463
	2 = 21–25	1604(78%)	451	2055
	3 = ≥ 26	354(67.7%)	169	523
Birth order	1	0(0.0%)	1323	1323
	2–4	4157(100.0)	0	4157
	5–7	2690(100.0)	0	2690
	8+	977(100.0)	0	977
Child sex	Male	4005(85.2%)	698	4703
	Female	3819(86%)	625	4444
Survival status of the index child	Dead	453(86.6%)	70	523
	Alive	7371(85.5%)	1253	8624
Breastfeeding status	No	2717(86.3%)	433	3150
	Yes	5107(85.2%)	890	5997
Contraceptive use	No	5933(88.9%)	738	6671
	Yes	1891(76.4%)	585	2476
Mass media	No	5870(87.3%)	853	6723
	Yes	1954(80.6%)	470	2424
Knowledge of ovulatory cycle	No	3286(89.1%)	402	3688
	Yes	4538(83%)	921	5459

According to Table 1, 5856 (64%) of the women and 4067 (44.5%) of the husbands have not had any formal education, 2288 (25%) of the women and 3220 (35.2%) of the husbands have at least primary level education, 1003 (11%) of the women and 1785 (19.5%) of the husbands have a formal educational level of at least secondary school and above. About 5775 (63.2%), 1466 (16%) and 1906(20.8%) of the women belonged to poor, middle and rich households, respectively. The output shows that 1106 (12%) of the women had first birth before the age of 16, while 5463(59.7%) and 2055(22.5%) of the women had their first birth in the ages 16–20, 21–25 years respectively. 523 (5.7%) of the women had their first birth above 26 years. 7465 (81.6%) were rural dwellers, while 1682(18.4%) were urban.

The median survival time of time-to-birth interval for rural women, is 33 months with 95% CI [32.62, 33.68] is less than that of urban women, 34 months with its 95% CI [33.23, 34.77] and its *P* value is 0.0213. The median survival time of time-to-birth interval for contraceptive users i.e., 38 months with [37.13, 38.87], was greater than that of non-users (32 months) with 95% CI [31.585, 32.415] and its *P* value is < 0.001. Women who have a job had a median survival time of 34 months with 95% CI [33.48,34.52] which was greater than that of jobless women (33 months) with 95% CI [32.54,33.46] based on *P* value is < 0.001. The median time of birth intervals for illiterate (who can’t read and write) women was 30 months with 95% CI [29.58, 30.42] which is less than that of women having primary education (36 months) with 95% CI [35.06, 36.94] and secondary school and above education (79 months) with 95% CI [76.96, 81.04] and its *P* value is < 0.001. The overall mean and median survival time of birth intervals for Ethiopian women is 37 months with 95% CI [40.20, 41.41] and 32 months with 95% CI [32.66, 33.34], respectively.

**Fig. 1:**
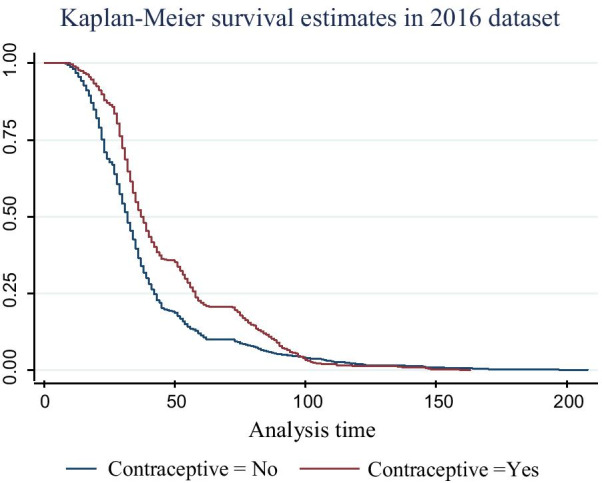
Survival curve of women by contraceptive use

The survival plot of time-to-birth interval by contraceptive use is given in Fig. 1. This plot showed that as compared to non-user contraceptive women, women who use contraceptive better timing of their birth interval. The log rank test also revealed that contraceptive use had significant association with time-to-birth interval of women (*P* < 0.001).

**Fig. 2:**
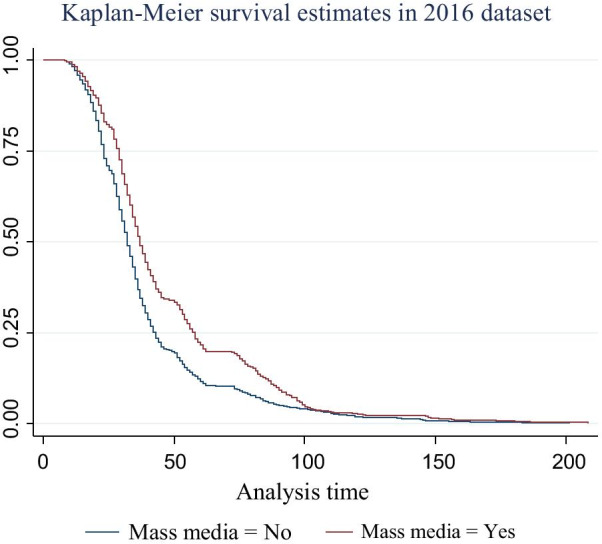
Survival curve of women’s by access to mass media

The survival plot of time-to-birth interval of women by access to mass media is shown in Fig. 2. The plot indicates that the probability of giving birth after birth is similar both for women who had access to mass media and who hadn’t. However, the difference becomes visible towards the middle of the curve and gets closer towards the end. At the middle of the curve, the survival plot of the birth interval for women having access to mass media had better birth interval timing than women who didn’t have mass media access.

### Determinants of successive birth interval accelerated failure time (AFT) model

For time-to-birth interval data, multivariable AFT models of Weibull, log-logistic, and log-normal distribution were fitted including all the covariates significant in the univariate analysis at 10% level of significance. To compare the efficiency of different models, Akaki information criteria (AIC) which is the most common applicable criterion to select model was used. Based on the AIC, the model having a minimum AIC value was selected. Accordingly, Log-logistic AFT model (AIC = 58,784.19) was the best one of the time-to-birth interval data set of the given alternative among the covariates significant in the univariate analysis.

Covariates insignificant in the multivariate analysis were removed from the model using backward elimination technique. Accordingly, Knowledge of ovulatory cycle, family’s access to mass media, contraceptive and employment status of women were excluded. The final model kept the covariate age of women at first birth, family wealth index, women’s marital status, religion and educational level of both spouses.

As shown in Table 2, using age at first birth of less than or equal to 15 as reference, in the log-logistic AFT model, when the effect of other factors is kept constant, the estimated acceleration factor for the age ranges at first birth of 16–20, 21–25 and greater than or equals to 26 was 1.04, 1. 2 and 1.4 with 95% CI [1.03, 1.07], [1.17, 1.23] and [1.32, 1.43], respectively. This indicates that compared with the women whose age was less than or equals to 15, women whose age at first birth was 16–20, 21–25 and ≥ 26 had longer birth interval. Using women who have orthodox religion as reference, the acceleration factor for Muslim women and other follower was 1.04 and 1.11, respectively. This result indicates that Muslim women and other follower had longer survival of time-to-birth interval than orthodox women based on the significance value which is less than the level of significance value (α = 0.05).

**Table 2:** Summary result of the final log-logistic AFT model

Covariate	Categories	Estimate $(\hat{\beta })$	$\varPhi$	95% CI	SE $(\hat{\beta })$	*P* value
Wealth index	Poor	Ref	Ref	Ref	Ref	Ref
	Middle	0.0283	1.029	[1.006,1.05]	0.01132	0.0125*
	Rich	0.0293	1.029	[1.006,1.05]	0.01048	0.0052*
Religion	Orthodox	Ref	Ref	Ref	Ref	Ref
	Catholic	0.0648	1.1	[0.98, 1.16]	0.04144	0.12
	Protestant	0.0052	1.01	[0.98, 1.03]	0.01123	0.599
	Muslim	0.042	1.04	[1.02, 1.06]	0.0104	< 0.001*
	Traditional	0.0229	1.02	[0.96,1.07]	0.03408	0.50
	Other	0.106	1.11	[1.03,1.28]	0.03829	0.0056*
Age at first birth	≤ 15	Ref				
	16–20	0.0488	1.04	[1.03, 1.07]	0.01026	< 0.001*
	21–25	0.185	1.2	[1.17, 1.23]	0.01179	< 0.001*
	≥ 26	0.317	1.4	[1.32,1.43]	0.01848	< 0.001*
Marital status	Single	Ref				
	Married	0.0398	1.04	[1.01,1.06]	0.01242	0.0014*
	Divorced	0.0942	1.1	[1.06,1.14]	0.01739	< 0.001*
	Windowed	0.0704	1.07	[1.03,1.11]	0.01883	< 0.001*
	Separated	0.014	1.01	[0.97,1.05]	0.02057	< 0.001*
Husbands education	No-education	Ref	Ref	Ref	Ref	Ref
	Primary	0.389	1.48	[1.45,1.5]	0.0077	< 0.001*
	Secondary and above	1.02	2.77	[2.71, 2.8]	0.0118	< 0.001*
Women education	No-education	Ref				
	Primary	0.0015	1.002	[0.98,1.02]	0.0067	0.863
	Secondary and above	0.108	1.11	[1.08,1.15]	0.015	< 0.001*

$\varPhi$ indicates acceleration factor, *significant at 5% level, 95% CI, confidence interval for acceleration factor; SE ($\hat{\beta }$), standard error for $\widehat{ \beta }$; Ref., reference

Using uneducated women, who cannot read and write, as reference, the acceleration factors for women attending primary education and secondary school and above level of education are estimated to be 1.002 and 1.11, respectively. This implies that women attending secondary school and above level of education had longer survival of time-to-birth interval, while for women attending primary level education, it was insignificant. Likewise, using uneducated husbands as reference, the acceleration factor for husbands attending primary education and secondary school and above level of education are estimated 1.5 and 2.8 with 95% CI (1.45, 1.5) and (2.71, 2.8) respectively and *P* value was less than the level of significance which implies that husbands attending secondary school and above level of education and primary level of education had longer survival of time-to-birth interval.

Using single marital status as reference, the acceleration factor for women whose marital status are married, divorced, widowed and separated are estimated to be 1.04, 1.1, 1.07 and 1.01 respectively. Moreover, using singles as reference, women whose marital status is married, divorced, widowed, and separated have longer survival of time to birth interval. The acceleration factors for families whose wealth index are middle and rich are 1.029 and 1.029 respectively. It also indicated that compared to families of poor wealth index, middle and rich wealth index families had longer survival of time to birth interval.

To identify baseline distribution and associated risk factors and to analyze the survival of time to birth interval, three AFT models were fitted and compared. The log-Logistic AFT model was selected based on AIC value. The main focus of this study was to investigate risk factors associated with the survival of time to birth interval using parametric shared frailty models. For the data on time-to-birth interval, the three parametric baseline distributions with Gamma frailty and inverse Gaussian distribution were fitted by using regional states of the women as frailty term. The effect of random component (frailty) was significant for both log-normal-gamma shared frailties, log-logistic gamma shared frailty models, and Weibull-gamma shared frailty model. The AIC value for all parametric frailty models is summarized and the given log-logistic gamma shared frailty model had smaller AIC value (58,793). This indicates that log-logistic gamma shared frailty model is a more suited and efficient model to describe time to birth interval dataset.

### Log-logistic inverse gaussian frailty model results

This model is the same as the log-Logistic AFT model discussed previously, except that a frailty component has been included. The frailty in this model is assumed to follow an inverse Gaussian distribution with mean 1 and a variance equal to theta (θ). The estimated value of theta ($\theta$) is 0.0013. A variance of zero ($\theta$ = 0) would indicate that the frailty component does not contribute to the model. A likelihood ratio test for the hypothesis $\theta$ = 0 shown in at the bottom of Table 3 indicating a chi-square value of 9616.02 with thirty two degrees of freedom resulted in a highly significant *P* value of < 0.001. This indicates that the frailty component had significant contribution to the model but the associated Kendall’s tau ($\tau$), which measures dependence with in clusters (region) is estimated to be 0.00065. The estimated value of the shape parameter in the log-logistic inverse Gaussian frailty model is 3.00 ($\rho = 3.00$). This value showed the shape of uni-modal hazard function because the value is greater than a unit implies that it increases up to its maximum point and then begins to decrease.

**Table 3:** Summary result of the final log-logistic inverse Gaussian shared frailty model

Covariate	Categories	Estimate ($\hat{\beta }$)	$\varPhi$	95%CI	SE ($\hat{\beta }$)	*P* value
Wealth index	Poor	Ref	Ref	Ref	Ref	Ref
	Middle	0.0339	1.03	[1.01,1.06]	0.01200	0.0048*
	Rich	0.0351	1.04	[1.01,1.06]	0.01125	0.0018*
Religion	Orthodox	Ref	Ref	Ref	Ref	Ref
	Catholic	0.0705	1.07	[1.08,1.16]	0.04191	0.092*
	Protestant	0.0152	1.012	[0.99,1.04]	0.01374	0.27
	Muslim	0.0382	1.04	[1.001,1.06]	0.01242	0.002*
	Traditional	0.0260	1.03	[0.96,1.09]	0.03499	0.46
	Other	0.0119	1.012	[0.92,1.09]	0.03915	0.003*
Age at first birth	≤ 15	Ref				
	16–20	0.049	1.05	[1.03,1.07]	0.01027	< 0.001*
	21–25	0.185	1.20	[1.17,1.23]	0.01181	< 0.001*
	≥ 26	0.318	1.37	[1.01,1.43]	0.01848	< 0.001*
Marital status	Single	Ref				
	Married	0.0384	1.04	[1.01,1.06]	0.01248	< 0.001
	Divorced	0.0955	1.10	[1.06,1.14]	0.01748	< 0.001
	Windowed	0.0713	1.074	[1.03,1.11]	0.01891	< 0.001
	Separated	0.116	1.12	[1.08,1.17]	0.02085	< 0.001*
Husbands education	No-education	Ref				
	Primary	0.389	1.48	[1.45,1.5]	0.0077	< 0.001*
	Second and above	1.02	2.77	[2.7,2.9]	0.01185	< 0.001*
Women education	No-education	Ref				
	Primary	0.00204	1.002	[0.99,1.02]	0.0083	0.81
	Second and above	0.108	1.114	[1.07,1.16]	0.0191	< 0.001*
$\theta$ = 0.0013	$\tau$ = 0.00065	$\uplambda = 0.17$	$\rho = {3}.00$			AIC = 58,792.14

Likelihood ratio test of $\theta = 0:{\text{chi - square}}$ = 9616.02 *P* value < 0.001*

$\varPhi$ indicates acceleration factor, *significant at 5% level, 95% CI,: confidence interval for acceleration factor; SE ($\hat{\beta }$), standard error for $\widehat{ \beta }$; Ref., reference

From Table 3, the confidence interval of the acceleration factor for all significant categorical covariates does not include one at 5% level of significance. This shows that they are significant factors for determining the survival of time-to-birth interval among women in Ethiopia. However, taking the uneducated women as reference and based on the covariate, women who have primary level of education is not significant at (*P* value = 0.81, ϕ = 1.002, 95% CI = 0.99, 1.02).

The age of women at first birth was statistically significant in determining the time-to-birth interval of Ethiopian women. The acceleration factor for all categories of age at first birth was 1.05, 1.20 and 1.37, with 95% confidence interval [1.03, 1.07], [1.17, 1.23] and [1.01, 1.43]) respectively, yielding a significant *P* value for all age categories was less than the level of significant (α = 0.05). As compared to women whose age group was less than or equal to 15, all the categories of age of women at first birth 16–20, 21–25, and ≥ 26 respectively have shorter birth interval. Additionally, the confidence interval did not include one indicating that the age of women at first birth was statistically significant for the survival of time-to-birth interval. Using orthodox religion of women as reference, the acceleration factors for Muslim women were 1.02, 1.012 and 1.07. This indicates that Muslim women and other follower had longer survival of time-to birth interval than women who follow orthodox religion, respectively.

Using uneducated women as reference, the acceleration factors for women attending primary level of education and secondary school and above level of education are estimated to be 1.002 and 1.114 respectively. This implies that women have secondary school and above level of education have longer survival of time-to-birth interval. Among women in their primary level of education however, the interval was not significant. Taking uneducated husbands as reference, the acceleration factors for husbands attending primary level of education and secondary school and above level of education are estimated to be 1.48 and 2.77 respectively. This implies that husbands attending secondary school and above level of education and primary level of education have longer survival of time-to-birth interval. Taking single marital status as reference, the acceleration factor for the marital status of women such as married, divorced, widowed and separated are estimated to be 1.04, 1.10, 1.074 and 1.12, respectively. This indicates that compared to the single women, women who were married, divorced, widowed and separated have longer survival of time-to-birth interval. The acceleration factor of families belonging to middle and rich wealth index were 1.03 and 1.04, respectively which imply that families who have middle and rich wealth index have longer survival of time to birth interval.

### Comparison of log-logistic AFT and log-logistic inverse Gaussian frailty model

From Table 4, it can be seen that the results from the Log-Logistic AFT and Log-Logistic Inverse Gaussian frailty model are quite similar though not identical. AIC was used to compare the efficiency of the models. Table 4 shows that compared to log-logistic shared inverse Gaussian frailty model (AIC = 58,792.16), the log-logistic AFT model has a lower AIC (58,784.19) indicating that log- logistic AFT model fitted the survival of time-to-birth interval data better than the log-logistic shared inverse Gaussian frailty model, which took the clustering effect in to account. The estimated value of coefficients of the covariate are altered with the inclusion of the frailty component, and the confidence interval for the acceleration factor is slightly narrower for log-logistic inverse Gaussian frailty model. In general, for modeling time-to-birth interval dataset, log-logistic AFT is preferred over Log-logistic inverse Gaussian shared frailty model. The graphical method of model checking was also used to strengthens the decision made by AIC value that log-logistic baseline distribution is appropriate for the given data.

**Table 4:** Comparison of Log-logistic AFT and inverse Gaussian Frailty model

Covariate	Categories	Log-logistic inverse Gaussian frailty			Log-logistic AFT		
		Estimate $\widehat{\left( \beta \right)}$	$\varPhi$	95% CI	Estimate $\widehat{\left( \beta \right)}$	$\varPhi$	95% CI
Wealth index	Poor	Ref	Ref	Ref	Ref	Ref	Ref
	Middle	0.0339	1.03	[1.01,1.06]	0.0283	1.029	[1.006,1.05]
	Rich	0.0351	1.04	[1.01,1.06]	0.0293	1.029	[1.006,1.05]
Religion	Orthodox	Ref	Ref	Ref	Ref	Ref	Ref
	Catholic	0.0705	1.07	[1.08,1.16]	0.0648	1.1	[0.98,1.16]
	Protestant	0.0152	1.012	[0.99,1.04]	0.0052	1.01	[0.98,1.03]
	Muslim	0.0382	1.04	[1.001,1.06]	0.042	1.04	[1.02,1.06]
	Traditional	0.0260	1.03	[0.96,1.09]	0.0229	1.02	[0.96,1.07]
	Other	0.0119	1.012	[0.92,1.09]	0.106	1.11	[1.03,1.28]
Age at first birth	≤ 15	Ref	Ref	Ref	Ref	Ref	Ref
	16–20	0.049	1.05	[1.03,1.07]	0.0488	1.04	[1.03,1.07]
	21–25	0.185	1.20	[1.17,1.23]	0.185	1.2	[1.17,1.23]
	≥ 26	0.318	1.37	[1.01,1.43]	0.317	1.4	[1.32,1.43]
Marital status	Single	Ref	Ref	Ref	Ref	Ref	Ref
	Married	0.0384	1.04	[1.01,1.06]	0.0398	1.04	[1.01,1.06]
	Divorced	0.0955	1.10	[1.06,1.14]	0.0942	1.1	[1.06,1.14]
	Windowed	0.0713	1.074	[1.03,1.11]	0.0704	1.07	[1.03,1.11]
	Separated	0.116	1.12	[1.08,1.17]	0.014	1.01	[0.97,1.05]
Husbands education	No-educa	Ref	Ref	Ref	Ref	Ref	Ref
	Primary	0.389	1.48	[1.45,1.5]	0.389	1.48	[1.45,1.5]
	Second and above	1.02	2.77	[2.7,2.9]	1.02	2.77	[2.71,2.8]
Women education	No-educa	Ref	Ref	Ref	Ref	Ref	Ref
	Primary	0.00204	1.002	[0.99,1.02]	0.0015	1.002	[0.98,1.02]
	Second and above	0.108	1.114	[1.07,1.16]	0.108	1.11	[1.08,1.15]
AIC = 58,792.16						AIC = 58,784.19	

$\varPhi$ Indicates acceleration factor, 95% CI, confidence interval for acceleration factor; SE($\hat{\beta }$), standard error for $\widehat{ \beta }$; Ref., Reference; AIC, Akaka’s information criteria

## Discussion

The main goal of the study was to model the determinants of time-to-birth interval of women in Ethiopia and to know the trend of the two data set using AFT and parametric shared frailty models based on three baseline distributions. The aim of frailty model is not only to account heterogeneity subjects among different regions but also to measure the dependence or correlation within the same region. Gamma distribution is selected for the frailty term due to its mathematical tractability and its flexibility in case of hazard function [4, 25] and strength of the AIC value.

This finding indicates that the husband’s education level is important covariate for determining birth interval of women. The result showed that compared to uneducated husbands, husbands whose level of education is primary or secondary school have women who have a longer birth interval. Compared with husbands who are uneducated, husbands who have primary and secondary school level of education decelerate the survival of birth interval 1.48 and 2.78 times respectively. This finding is consistent with the results of the study conducted by Hidayat et al. [9] in Indonesia.

Another important factor affecting the survival of birth interval is the educational level of women. This finding shows that women who have secondary school and above level of education have a longer survival of birth interval than uneducated women. Consequently, they decelerate the survival of birth interval 1.11 times that of the uneducated. This finding is similar with results of studies by [1, 8].

Religion is a covariate affecting the survival of time to birth interval of women. Muslim and other religion follower women have longer survival of time to birth interval than women orthodox in their religion. Muslim women decelerate the survival of time to birth interval 1.02 times that of women who are orthodox followers in their religion. On the other hand, women who are follower of other religions decelerate the survival time to birth interval 1.09 times that of women whose religion is orthodox. This finding is consistent with studies conducted in Ethiopia by [7, 18]. Age at first birth of child was a significant predictor of birth interval. Women aged 16–20 years elongate the survival of birth interval of women by about 4.9% while those aged less or equal to 15, and aged 21–25 or more lengthened the survival of birth interval by about 18.5%, 31.8% respectively. Older women were more likely to achieve the desired number of children. Another reason is that women’s role in fixing the number of children differ because of different shorter and longer survival times. Studies from southern Ethiopia and northern Iran supported this finding by [26].

Another important factor affecting the survival of birth interval of women is marital status of women. This finding showed that women whose marital status was married, divorced, widowed, and separated had longer survival of birth interval than single women. The acceleration factor for women that decelerate the survival of birth interval of women who are married, divorced, widowed and separated in their marital status are respectively 1.04, 1.07,1.05 and 1.09 times that of women’s whose marital status are single which is confirmed with the finding of [2]. Wealth index is found significantly influence the timing of birth intervals as women with a medium wealth index had a birth interval that is longer than women with a lower index of wealth. Similarly, women belonging to higher wealth index had a birth interval that is longer than women belonging to a lower wealth index. Women who have medium wealth index have a birth interval that is 1.03 times lower than that of women belonging to a lower socio-economic stratum, and a risk of having next birth that is 1.04 times lower than women belonging to higher wealth index. A study conducted by [14] in Uganda and Zimbabwe also supported the findings of the present study.

## Conclusions

This study aimed at modeling the determinant of time-to-birth interval among women who have at least one child in Ethiopia drawing on two datasets of central statistics agency and using parametric AFT model. Keeping other factors constant, women who have longer birth interval tend to have smaller family size when compared with women who have shorter inter-birth intervals. Differences in exposure to the risk of pregnancy and in the length of time between births when women are exposed to child bearing causes differences in fertility levels between different populations or between groups within the same populations. Helping women achieve healthy pregnancy and safe birth is one of the priorities of every country’s family planning program.

This study attempted to investigate determinants of successive birth interval in Ethiopia. The findings indicate that age of women at first birth, educational level of women and husbands, religion, marital status, and family wealth index significantly affect length of birth interval of Ethiopian women. Modernization that induces cultural changes and new sexual and reproductive orientations in the youth led to emergent patterns of marriage and fertility that subsequently affect the length of successive birth interval.

Longer successive birth interval is likely to reduce the total fertility rate of a woman. Hence, given the prevalence of low level of education among women, improving the socio-economic status of women adjust their fertility and improves maternal and child wellbeing through well-spaced birth interval.

## Data Availability

This study used Ethiopian Demographic and Health Survey (EDHS) data which is a secondary data obtained from Ethiopian Central Statistical Agency. The data is freely available after simple registration and explaining the purpose of accessing the data at https://www.dhsprogram.com/data/.

## Abbreviations

AFT: Accelerated failure time
AIC: Akaike information criterion
EDHS: Ethiopian Demographic Health Survey
CI: Confidence interval
